# Case Report: Cholecystitis and gastric perforation caused by a fishbone and treated by laparoscopic surgery

**DOI:** 10.3389/fmed.2025.1523249

**Published:** 2025-03-19

**Authors:** Xuemin Li, Junfeng Cheng, Yonghang Wang, Zhijun Chen, Zewei Chen, Chao Ren

**Affiliations:** ^1^Department of Hepatobiliary and Pancreatic Surgery, The Affiliated Jinhua Hospital of Zhejiang University School of Medicine, Jinhua, China; ^2^Department of General Surgery, Hengdian Wenrong Hospital, Jinhua, China

**Keywords:** cholecystitis, gastric perforation, fishbone, laparoscopic surgery, treatment

## Abstract

Rare cases of swallowed fish bones leading to cholecystitis and gastric perforation have been reported. Here, we present the case of a 71-year-old male patient who experienced 10 days of right upper quadrant pain after eating fish. Laparoscopic repair of gastric perforation and cholecystectomy was performed and successfully removed a fishbone of 3 cm in length from the region between the gallbladder cavity and the gastric antrum. The patient was 10 days after surgery and recovered well. No sign of recurrence was observed at the 3-month follow-up.

## Introduction

The incidence of penetration of the digestive tract by foreign bodies has been estimated to be less than 1%, with migration to adjacent organs being an exceptionally rare complication. The most common sites of gastrointestinal (GI) perforation are the stomach and duodenum, potentially leading to various complications such as liver abscess (1), liver actinomycosis (2), and portal vein thrombosis (3). The foreign bodies involved are typically sharp and elongated, such as toothpicks, fish bones, and chicken bones (4, 5). Duodenal reflux through the plica spiralis usually prevents the foreign body from reaching the gallbladder. The detection of small radiolucent fish bones remains a challenge, particularly in patients with atypical symptoms, and can often result in initial misdiagnosis and delayed treatment if a detailed medical history has not been obtained. A review of the literature shows that there are few reports of fish bone-related cholecystitis to date (6–9), with even fewer reports describing concurrent gastroduodenal injury. Kunizaki et al. (7) reported a case of cholecystitis caused by a fish bone that penetrated the gallbladder without causing peritonitis and was treated with laparoscopic cholecystectomy. Here, a rare case of a 71-year-old male with simultaneous cholecystitis and gastric perforation after swallowing a fishbone highlights the importance of abdominal computed tomography (CT) scans, the collection of a detailed medical history, and laparoscopic surgery in achieving prompt diagnosis and intervention.

## Case presentation

**FIGURE 1:**
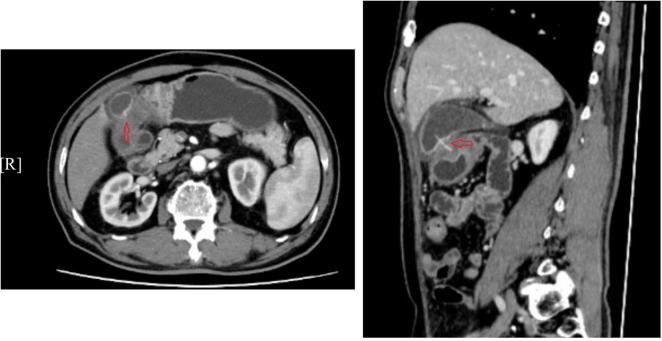
Abdominal CT scan showing a 3 cm curvilinear hyperdense foreign body (red arrow) positioned between the gallbladder cavity and gastric antrum, with surrounding inflammation.

**FIGURE 2:**
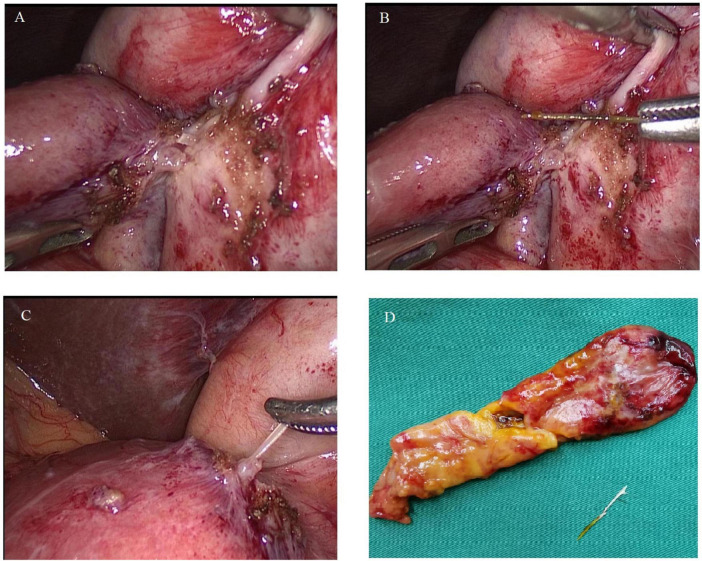
**(A)** Fish bone penetrating from the gallbladder into the gastric antrum, with adjacent inflammation. **(B)** Fish bone approximately 3 cm in length extracted from the gallbladder. **(C)** Fish bone extracted from the gastric antrum. **(D)** Surgical specimen displaying the gallbladder and the retrieved fish bone.

A 71-year-old man with a background of myocardial infarction was admitted to the hospital after experiencing 10 days of right upper quadrant, intermittent, and endurable pain. Before hospital admission, the patient had received only anti-infection and symptomatic treatment without surgery, which failed to relieve the right upper abdominal pain. When obtaining a detailed medical history, the patient reported a history of myocardial infarction and heart failure, and recalled eating fish 10 days before. Physical examination showed right upper quadrant tenderness without localized peritonitis. Laboratory findings revealed a total bilirubin level of 61.2 μmol/L (normal < 23 μmol/L) and a C-reactive protein value of 21.64 mg/dL (normal < 10 mg/dL) while other laboratory findings were within normal limits. Abdominal CT examination indicated the presence of a 3 cm curvilinear hyperdense foreign body between the gallbladder cavity and the gastric antrum, leading to the suspicion of gastric perforation caused by a fish bone without liver abscess (Figure 1). During surgery, a fish bone was found within the gallbladder, accompanied by surrounding inflammation and edema of the gallbladder wall without obstruction of the cystic duct; after exposure of the tail of the fishbone in the gastric antrum, it was cut with scissors and removed (Figure 2). Laparoscopic repair of the gastric perforation and cholecystectomy took approximately 1 h and was successful. The patient was discharged 10 days after surgery and recovered well, with removal of the drainage pipe and laboratory findings within normal limits. At the 3-month follow-up examination, the patient was found to have recovered with no recurrence of the right upper abdominal pain or other surgical complications, such as gastric leakage.

## Discussion

Acute cholecystitis is caused primarily by gallbladder lithiasis and presents commonly with right upper quadrant pain. It has been reported that acalculous cholecystitis accounts for only approximately 10% of acute cholecystitis cases, while cholecystitis with digestive tract perforation caused by swallowing a bone is highly unusual (10). In contrast to the case reported by Kunizaki et al. (7) in which the fish bone fortunately caused no gastric perforation and liver abscess, in the present case, swallowing a fish bone led to cholecystitis and gastric perforation, which were treated successfully by removal of the bone and repair of the perforation.

The methods used for the detection of swallowed foreign bodies primarily include CT scans, B ultrasound, and endoscopy (11, 12). Ultrasonography has limited value in the diagnosis of foreign bodies in the digestive tract and the detection of inflammation at the site of perforation (13). Szanto et al. (13) considered that enhanced CT was the first choice for diagnosing complications caused by fish bones, while Hainaux et al. (14) in an analysis of CT images, was successful in predicting GI perforation in 73 of 85 cases, indicating the accuracy and value of CT in the diagnosis of GI perforation. From our experience in this case, we consider that ultrasonography is more valuable than enhanced CT in the differential diagnosis of acalculous cholecystitis, while enhanced CT is helpful in identifying the specific location of the bone, excluding other causes of acute abdomen, and drawing up an appropriate treatment strategy.

Previously, due to the rarity of the condition, it was difficult to develop standard diagnostic and treatment guidelines for simultaneous cholecystitis and gastric perforation caused by swallowing bones, and thus undertake appropriate therapeutic decision-making. Several cases have been reported where fish bones have caused cholecystitis and have been treated with laparoscopic surgery (7, 8, 15). Tong et al. (6) described a case of pyloric perforation secondary to fish-bone ingestion that mimicked acute cholecystitis, where the foreign body was removed effectively using endoscopy with no complications of mucosal damage or bleeding. Azab et al. (16) also reported endoscopic removal of gastric-perforating fishbone, with closure of the defect using a scope clip. If perforation is limited to the gastric wall, endoscopic extraction and clipping may be possible (17–19). However, in the present case, we decided against using endoscopic treatment for closing the fistula with titanium clips as the hole made by the fish bone resulted in complete perforation between the gallbladder cavity and gastric antrum. The risk of bone residues or missed injuries necessitates thorough intraoperative exploration. There are thus advantages to laparoscopic surgery or laparotomy. The choice of endoscopic management or laparoscopic approach to treat cholecystitis and gastric perforation caused by fish bones thus requires further investigation in the future.

While the collection of a detailed medical history is of great importance, many patients do not recall eating fish and swallowing a bone. Information on the patient’s diet, however, can provide suggestions as to the nature of the foreign body, enabling guidance of further treatment. Laparoscopy is effective not only for an accurate diagnosis but also for prompt treatment (7, 15). In summary, despite the age of the patient and his background of myocardial infarction and heart failure, we succeeded in performing laparoscopic surgery early, avoiding morbidity and mortality. The information on the patient’s diet of fish was of significant help in this respect. It is also important to avoid slippage of the fishbone under laparoscopy to prevent difficulties in location if it passes into the digestive tract.

## Data Availability

The original contributions presented in this study are included in this article/supplementary material, further inquiries can be directed to the corresponding author.
